# *Eucalyptus* and Native Broadleaf Mixed Cultures Boost Soil Multifunctionality by Regulating Soil Fertility and Fungal Community Dynamics

**DOI:** 10.3390/jof10100709

**Published:** 2024-10-11

**Authors:** Huaxiang Wang, Dian Tian, Jizhao Cao, Shiqi Ren, Yuanli Zhu, Huili Wang, Lichao Wu, Lijun Chen

**Affiliations:** 1 Key Laboratory of Soil and Water Conservation and Desertification Combating of Hunan Province, Central South University of Forestry and Technology, Changsha 410004, China; 19965052865@163.com (H.W.); 13409941601@163.com (D.T.); wulichao@sina.com (L.W.); 2 Key Laboratory of Cultivation and Protection for Non-Wood Forest Trees, Ministry of Education, Central South University of Forestry and Technology, Changsha 410004, China; 3 Guangxi Zhuang Autonomous Region Forestry Research Institute, Nanning 530002, China; jizhaocao@163.com (J.C.); rensq_20220901@163.com (S.R.); wanghuili6@163.com (H.W.); 4 Qipo State-Owned Forest Farm of Guangxi Zhuang Autonomous Region, Nanning 530225, China; zylsu37@163.com

**Keywords:** soil multifunctionality, *Eucalyptus* mixed-culture native broadleaf, fungal community, co-occurrence network

## Abstract

The growing recognition of mixed *Eucalyptus* and native broadleaf plantations as a means of offsetting the detrimental impacts of pure *Eucalyptus* plantations on soil fertility and the wider ecological environment is accompanied by a clear and undeniable positive impact on forest ecosystem functions. Nevertheless, the question of how mixed *Eucalyptus* and native broadleaf plantations enhance soil multifunctionality (SMF) and the mechanisms driving soil fungal communities remains unanswered. In this study, three types of mixed *Eucalyptus* and native broadleaf plantations were selected and compared with neighboring evergreen broadleaf forests and pure *Eucalyptus* plantations. SMF was quantified using 20 parameters related to soil nutrient cycling. Partial least squares path modeling (PLS-PM) was employed to identify the key drivers regulating SMF. The findings of this study indicate that mixed *Eucalyptus* and native broadleaf plantations significantly enhance SMF. Mixed *Eucalyptus* and native broadleaf plantations led to improvements in soil properties (7.60–52.22%), enzyme activities (10.13–275.51%), and fungal community diversity (1.54–29.5%) to varying degrees compared with pure *Eucalyptus* plantations. Additionally, the mixed plantations exhibit enhanced connectivity and complexity in fungal co-occurrence networks. The PLS-PM results reveal that soil properties, fungal diversity, and co-occurrence network complexity directly and positively drive changes in SMF. Furthermore, soil properties exert an indirect influence on SMF through their impact on fungal diversity, species composition, and network complexity. The findings of this study highlight the significant role of mixed *Eucalyptus* and native broadleaf plantations in enhancing SMF through improved soil properties, fungal diversity, and co-occurrence network complexity. This indicates that incorporating native broadleaf species into *Eucalyptus* plantations can effectively mitigate the negative impacts of monoculture plantations on soil health and ecosystem functionality. In conclusion, our study contributes to the understanding of how mixed plantations influence SMF, offering new insights into the optimization of forest management and ecological restoration strategies in artificial forest ecosystems.

## 1. Introduction

In the context of global climate change and ecological degradation, soil multifunctionality (SMF) has emerged as a crucial indicator of soil ecosystem service capacity [1]. SMF encompasses essential roles such as nutrient cycling, water regulation, carbon storage, and biodiversity preservation, which are crucial for agricultural production, environmental protection, and climate change mitigation [2]. In forest ecosystems, SMF is of particular significance. It provides nutrients and water for plant growth, promotes biodiversity, maintains ecosystem stability, and enhances carbon sequestration [3,4]. Despite the importance of SMF, research in this field has predominantly focused on agricultural and grassland ecosystems [5,6], with relatively less attention given to forests, which represent the largest terrestrial ecosystem on Earth. Given the threats posed by global warming and human activities to biodiversity and ecosystem functions, investigating the drivers of SMF in forests is essential. Such research can elucidate the functions and services of forest ecosystems, thereby providing scientific evidence for their conservation and sustainable use.

*Eucalyptus* is a crucial fast-growing timber species globally, renowned for its robust vitality, wide adaptability, and high economic value [7]. However, the extensive planting and continuous management of commercial *Eucalyptus* plantations has led to reduced biodiversity and depletion of soil nutrients, threatening both regional and global ecological and timber security [8]. Improper management practices may be a major driver of soil quality decline [9]. The formation of mixed forests by mixing with broadleaf tree species has proven to be an effective strategy for mitigating soil fertility degradation in intensive plantations [10]. Mixed forests can optimize spatial and resource utilization, regulate the microclimate, boost forest output, elevate species richness, and improve soil nutrient cycling and ecosystem functions [11]. Research indicates that combining *Eucalyptus* with nitrogen-fixing species can enhance soil quality [12]. However, not all regions are appropriate for cultivating nitrogen-fixing trees. In some regions, combining *Eucalyptus* with nitrogen-fixing species can enhance nitrogen utilization efficiency, organic carbon, nitrogen, phosphorus, and timber yield [13], but in other areas, it may result in lower stand productivity compared to pure *Eucalyptus* plantations [14]. Native broadleaf species, due to their unique ecological roles, are considered the best choice. Research has found that mixing *Eucalyptus* with native species results in higher soil quality, productivity, and understory biodiversity and more complex vegetation structures [15]. However, current studies on *Eucalyptus* mixed forests primarily concentrate on single functions like productivity, carbon distribution, nutrient cycling, and microbial community diversity, with few investigations into SMF in mixed *Eucalyptus* plantations [10,12]. Therefore, investigating the impacts of mixing *Eucalyptus* with native broadleaf species on SMF is essential for fully understanding and enhancing forest ecosystem roles and benefits, along with the sustainable development of *Eucalyptus* plantations.

A complex and diverse soil microbial community is essential for soil ecological processes and regulates the soil ecosystem’s response to human-induced disturbances and environmental changes [16]. The composition, structure, and function of soil microbial communities are highly responsive to alterations in the soil environment. Alterations in these communities can significantly impact SMF by affecting greenhouse gas emissions, as well as carbon and nutrient cycling, leading to broad and important environmental consequences [17,18]. Fungi, as a significant component of global biodiversity, are closely associated with plants through various processes such as nutrient absorption, organic matter decomposition, disease mechanisms, and predicting forest carbon storage [19]. Therefore, soil fungal community diversity may potentially drive forest SMF [20]. For instance, studies have found that the loss of fungal rather than bacterial diversity may lead to reduced soil functions related to nutrient cycling and climate regulation in northern forests [3]. However, the impact of soil microbes on ecosystem functions is often studied through their effects on specific ecological processes, potentially overlooking trade-offs or synergies between these processes [16]. Different microbial taxa form complex interrelationships through predation, symbiosis, and competition, which can be explored using co-occurrence network analysis [21]. Co-occurrence network analysis, utilizing metrics such as edges, nodes, and average degree, investigates interactions among microbial taxa, revealing complex ecological relationships influenced by resource availability and environmental heterogeneity [22]. Microbial network complexity has also been found to be a key factor influencing ecosystem functions and services, which is one of the main drivers of SMF changes [23]. Complex microbial networks can enhance ecosystem stability and resilience, thereby improving their ability to provide multiple ecosystem services [24]. However, it remains unclear how soil fungal diversity and its network complexity respond to changes brought about by mixing *Eucalyptus* with native broadleaf species and how these changes affect SMF. Therefore, further research in these areas is essential for the thorough comprehension and enhancement of forest ecosystem functions and services.

This study compares SMF and fungal community changes in mixed plantations of *Eucalyptus* and various native broadleaf species, exploring the relationship between soil fungal communities and SMF. We propose the following hypotheses: (a) mixed *Eucalyptus* and broadleaf plantations significantly enhance SMF; (b) mixed plantations significantly increase fungal community diversity and co-occurrence network complexity; and (c) soil nutrient content, fungal diversity, and co-occurrence network complexity are the primary drivers of SMF. These findings will provide a crucial understanding of the ecological benefits of *Eucalyptus* and broadleaf mixed plantations and their role in promoting sustainable forest management.

## 2. Materials and Methods

### 2.1. Study Area

This study selected the *Eucalyptus* plantation from the state-owned Qipo Forest Farm in Nanning City, Guangxi Zhuang Autonomous Region, China (21.85–22.10° N, 107.61–107.96° E), as the research object, which is the main planting area of *Eucalyptus* plantations in China. Situated south of the Tropic of Cancer, the area has a warm and humid monsoon climate with plenty of heat and precipitation and is very conducive to forestry production. The average temperature in the coldest month, January, is 12.8 °C, while the hottest months, July and August, have an average temperature of 28.2 °C. The annual average rainfall is 1304.2 mm, with an average relative humidity of 79%, indicating a hot and humid climate. The soil type in the study area comprised ferralsols based on the Food and Agriculture Organization classification system.

### 2.2. Experimental Design and Soil Sampling

This study was conducted in 2015 at the Qipo State Forest Farm in Guangxi, China. Areas with similar altitude, soil, and climate factors were selected to establish mixed forests of *Eucalyptus grandis* × *urophylla* and native broadleaf tree species. The study area was originally an evergreen broadleaf forest before planting. The mixed planting pattern adopts a banded mixed approach, with a *Eucalyptus*-to-broadleaf ratio of 2:1. The native broadleaf tree species involved in the mixture included *Castanopsis hystrix*, *Michelia macclurei*, and *Magnolia hypolampra*. Each forest stand covered an area of at least 10 hectares. Three mixed forest types (*Eucalyptus* mixed with *C. hystrix*, *M. macclurei*, and *M. hypolampra*) were designated MF1, MF2, and MF3, respectively. Additionally, the evergreen broadleaf forest and pure *Eucalyptus* forest planted in the same year were selected as controls, designated CK and PF, respectively, with the main tree species of CK being *Phoebe bournei*. The average diameter at breast height of the CK, PF, MF1, MF2, and MF3 plots was 20.37 cm, 16.93 cm, 21.53, 28.00 cm, and 20.20 cm, respectively, and the average height was 18.33 m, 18 m, 24 m, 20.33 m, and 20 m, respectively. In the study area, three standard plots (20 m × 30 m) were established as replicates for each treatment, ensuring they were spatially separated. The management practices and planting density were the same for each treatment. Each forest stand received an annual application of 0.8 t/ha of compound fertilizer, with an N:P:K ratio of 15:16:18.

In September 2022, surface soil samples (0–20 cm) were collected from 15 points within each standard plot using an “S” line sampling strategy. These samples were thoroughly mixed to form a composite sample. A total of 15 soil samples were collected (5 treatments × 3 replicates). Each fresh soil sample was subjected to a 5 mm mesh sieving process to remove any visible litter and gravel. Following this, the soil samples were divided into three portions. One of these was placed in a 10 mL sterile centrifuge tube and stored immediately at −80 °C to conduct DNA extraction and microbial analysis at a subsequent stage. The second portion was stored at 4 °C in preparation for conducting soil enzyme activity assays. The third portion underwent an air-drying process to conduct soil chemical property analysis subsequently.

### 2.3. Soil Properties and Enzymatic Activity Analysis

The pH of the soil was determined using a pH meter (Sartorius, Göttingen, Germany) following the extraction of water (soil-to-water ratio of 1:1.25). Soil organic carbon (SOC) was measured using the potassium dichromate–sulfuric acid colorimetric method [25]. Total nitrogen (TN) was determined using the Kjeldahl method [26]. The concentrations of NH_4_^+^-N and NO_3_^−^-N were determined by leaching with 2 mol·L^−1^ KCL and subsequent measurement of the resulting solution’s color with the indophenol blue method and UV spectrophotometry, respectively [27]. Total phosphorus (TP) and available phosphorus were determined using the sodium hydroxide fusion–molybdenum antimony colorimetric method [28] and the Mehlich 3 method, respectively [29]. Total potassium (TK) and available potassium (AK) were obtained using the alkali fusion flame spectrophotometry method [27].

Soil urease (Ure), invertase (Inv), and acid phosphatase (ACP) activities were cultured with urea, sucrose, and disodium phenyl phosphate, respectively [30]. Soil enzyme concentrations were expressed as μg of ammonia nitrogen, glucose, and l phenol produced per g of soil per day. Leucine aminopeptidase (LAP) was determined using the visible spectrophotometry method. Alpha-glucosidase (AG), β-D-glucosidase (BG), cellobiohydrolase (CB), N-acetyl-glycosaminidase (NAG), and polyphenol oxidase (PPO) were used to determine the pNp-microplate method [31]. The corresponding enzyme activities were determined with p-nitrobenzene-α-D-glucopyranoside, nitrobenzene-β-D-glucopyranoside, p-nitrobenzene cellopyranobiose, p-nitrobenzene, and L-3, 4-dihydroxyphenylalanine as substrates. The enzyme activity is expressed in terms of the micromolar concentration of p-nitrophenol catalytically produced per gram of organic carbon per hour (μmol g^−1^ organic carbon h^−1^). Each sample was set as a standard group, a control group, and a blank group, incubated at 37 °C for 24 h; the absorbance of a Star 96 microplate was measured using a microplate reader (SpectraMax i3X03030923, Molecular Devices, Sunnyvale, CA, USA), and the concentration of the product in the standard group was calibrated using the control group and blank group.

### 2.4. DNA Extracted and High-Throughput Sequencing

Microbial DNA was extracted from 0.5 g soil samples using the E.Z.N.A. Soil DNA Kit (Omega Bio-tek, Norcross, GA, USA) following the methodology outlined by the manufacturer. The quantity and quality of the extracted DNA were assessed using a NanoDrop ND-1000 spectrophotometer (NanoDrop Technologies, Wilmington, DE, USA). For high-throughput Illumina sequencing, the fungal ITS1 region was amplified using the primer pair 5′-CTTGGTCATTAGGAAGTAA-3′ (ITS1F) and 5′-GCTGCGTTTCTCATCGATGC-3′ (ITS2R) [32]. Polymerase chain reaction (PCR) amplification was conducted on an ABI GeneAmp^®^ 9700 thermal cycler (Applied biosystem, Waltham, MA, USA), employing the following conditions: an initial denaturation at 95 °C for 5 min, followed by 30 cycles of 95 °C for 30 s. The cycling parameters were as follows: an initial denaturation at 95 °C for 5 min, followed by 30 cycles of 95 °C for 30 s, 55 °C for 30 s, and 72 °C for 45 s, with a final extension at 72 °C for 10 min. The reaction was then held at 10 °C until halted by the user. Subsequently, the resulting amplicons underwent separation on a 2% agarose gel and purification through the use of the AxyPrep DNA Gel Extraction Kit (Axygen Biosciences, Union City, CA, USA). Thereafter, the purified DNA underwent quantification utilizing a Quantus Fluorometer (Promega, Madison, WI, USA) before being subject to sequencing on a MiSeq PE300 platform (Illumina, San Diego, CA, USA).

Sequencing data were stored in FASTQ files, which included both the sequence reads and their quality metrics. Quality control and sequence clustering were conducted using QIIME 2 (QIIME, version 1.9.1) [33], resulting in the generation of amplicon sequence variants (ASVs). Fungal representative sequences were annotated by comparison with the UNITE v8.3 database [34] using the RDP Classifier [35]. Subsequently, Illumina sequencing data were employed to appraise soil fungal community diversity, composition, and network structure. Alpha diversity metrics, such as Shannon and Chao1, were calculated to assess diversity within samples. Beta diversity, based on Bray–Curtis distances, was utilized to contrast community composition between samples. Co-occurrence networks were constructed to analyze fungal community interactions and network topology.

### 2.5. Soil Multifunctionality

The average method was used to calculate SMF. In the average method, 20 soil ecosystem parameters related to carbon, nitrogen, and phosphorus cycling, as well as microbial diversity, were selected to quantify SMF. These parameters included pH, SOC, TN, TP, TK, NH_4_^+^-N, NO_3_^−^-N, AP, AK, Ure, ACP, Inv, LAP, AG, BG, CB, NAG, PPO, Fungal Shannon, and chao1 index. For each of the 15 samples, we normalized the values of these parameters. Normalization was performed by calculating the z-scores for each parameter. The z-score for a parameter is calculated by subtracting the mean value of that parameter across all samples and then dividing it by the standard deviation of that parameter [36]. The z-scores for each parameter within a sample were averaged to produce a composite score. This composite score represents the SMF index for that sample. Essentially, the average of the z-scores provides a single value that reflects the overall SMF, integrating multiple ecosystem functions into one metric [36].

### 2.6. Data and Statistical Analyses

One-way analysis of variance (ANOVA) and Spearman correlation tests were conducted using SPSS 23.0 (IBM SPSS Statistics) to evaluate the differences and correlations in soil properties, enzyme activities, microbial diversity, and SMF across different treatments (*p* < 0.05, 0.01, or 0.001) [37,38]. Principal coordinate analysis (PCoA) based on the ASV level of unweighted unifrac distance and analysis of similarities (Anosim) was performed using the “ape” and “vegan” packages in R to visualize the similarity or dissimilarity of samples in a low-dimensional space [39]. PERMANOVA was employed to evaluate the dissimilarities in the fungal community structure across different treatments [40]. The “randomForest” package and Spearman correlation analysis in R v4.3.2 were further used to investigate the relationships between soil properties, topological properties of fungal co-occurrence networks, enzyme activity, and SMF [41]. Redundancy analysis (RDA) analysis was further conducted using the “vegan” packages in R v4.3.2 to investigate the relationship between soil properties and the structure of fungal communities [39]. The “Sparcc”, “EasyStat”, “ggClusterNet”, “igraph”, “sna”, “network”, and “SpiecEasi” packages in R v4.3.2 were used to calculate alpha diversity (Shannon and richness), construct microbial co-occurrence networks, and calculate the topological properties of fungal networks (e.g., edge, connectance, average degree, average path length, average clustering coefficient, centralization degree, centralization betweenness, centralization closeness, negative ratio), and then the networks were visualized using Gephi 9.3.1 [42]. A partial least squares path model (PLS-PM) was conducted to determine the direct and indirect effects of soil properties and fungal communities on SMF using the “plspm” package in R v4.3.2 [41,43]. Soil properties are represented by soil pH, TP, TK, and AP. Fungi diversity is represented by fungi Shannon and Chao1 index. Fungi composition is represented by the relative abundance of Basidiomycota, unclassified, Ascomycota, and Mucoromycota. Fungi networks are represented by edge, connectance, average degree, average clustering coefficient, centralization degree, centralization betweenness, and centralization closeness. SMF is represented by the SMF index. According to the established criteria, the overall model fit was classified into the following categories: weak, moderate, and strong. These categories were based on the threshold values of the goodness-of-fit (GoF) index, which are as follows: 0.1, 0.25, and 0.36 [44]. Subsequently, the GoF was calculated for both the component-based and covariance-based PLS-PM.

## 3. Results

### 3.1. Soil Properties and Enzymatic Activities

**Figure 1:**
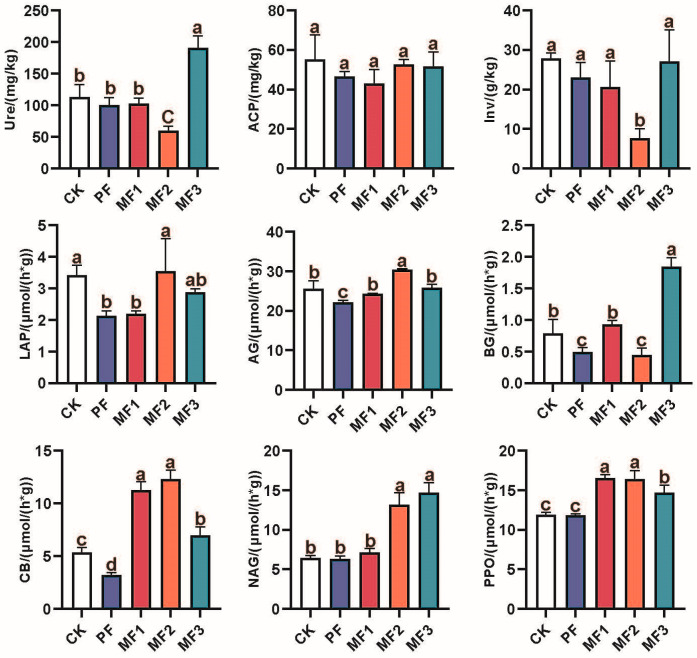
Differences in soil enzyme activities among different treatments. Different lowercase letters indicate significant variations among different treatments (*p* < 0.05). Ure: urease; ACP: acid phosphatase; Inv: invertase; LAP: leucine aminopeptidase; AG: alpha-glucosidase; BG: β-D-glucosidase; CB: cellobiohydrolase; NAG: N-acetylglucosamines; PPO: polyphenol oxidase; CK: evergreen broadleaf forest. PF: pure *Eucalyptus* forest. MF1: mixed *Eucalyptus* and *Castanopsis hystrix* forest. MF2: mixed *Eucalyptus* and *Michelia macclurei* forest. MF3: mixed *Eucalyptus* and *Magnolia hypolampra* forest.

Mixed *Eucalyptus* and broadleaf plantations significantly affected soil properties and enzyme activities (*p* < 0.05, Table 1 and Figure 1). The soil properties in different mixed plantations exhibited a range of variations compared to pure *Eucalyptus* plantations. The MF1 treatment demonstrated no significant changes (*p* > 0.05), whereas the MF2 treatment resulted in a significant increase in soil pH, NH_4_^+^-N, and NO_3_^−^-N contents. Additionally, the MF3 treatment led to a significant enhancement in SOC content with an observed range of 7.60% to 52.22% (*p* < 0.05). The changes in soil enzyme activity in different mixed plantations had different trends compared to that of pure *Eucalyptus*. In comparison to PF treatment, the MF1 treatment demonstrated a statistically significant increase in the activities of AG, BG, CB, and PPO, with a mean increase of 10.13% to 250.31%. The MF2 treatment resulted in a significant increase in the activities of LAP, AG, CB, NAG, and PPO, with a mean increase of 37.58% to 281.68%. The MF3 treatment also demonstrated a significant increase in the activities of Ure, LAP, AG, BG, CB, NAG, and PPO, with a mean increase of 17.05% to 275.51%.

### 3.2. Diversity and Species Composition of Soil Fungal Communities

**Figure 2:**
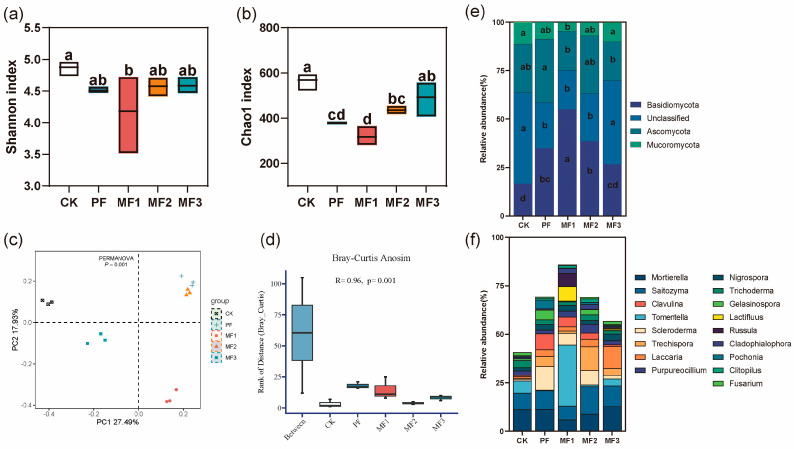
Differences in soil fungal diversity and species composition of different treatments. (**a**) Shannon index. (**b**) Chao1 index. (**c**) Beta diversity. (**d**) Anosim analysis. (**e**,**f**) Phylum- and genus-level species composition. Different lowercase letters indicate significant differences among different treatments (*p* < 0.05). The significance of the differences between groups in the PCoA analysis was tested by PERMANOVA (*p* < 0.05). The R-value is used to indicate whether there is a difference in genus between different groups, and the *p*-value is used to indicate whether there is a significant difference in Anosim analysis. The species composition at the genus level is that the average relative abundance between the treatments is greater than 1%. CK: evergreen broadleaf forest. PF: pure *Eucalyptus* forest. MF1: mixed *Eucalyptus* and *Castanopsis hystrix* forest. MF2: mixed *Eucalyptus* and *Michelia macclurei* forest. MF3: mixed *Eucalyptus* and *Magnolia hypolampra* forest.

Evergreen broadleaf forest soil fungal communities exhibit higher Shannon and Chao1 indexes compared to pure *Eucalyptus* plantations and mixed *Eucalyptus*–broadleaf plantations (Figure 2a,b). The mixed *Eucalyptus*–broadleaf plantations demonstrate a range of increases in soil fungal Shannon and Chao1 indices, with values varying between 1.54% and 1.76% for Shannon and between 14.24% and 29.50% for Chao1 (Figure 2a,b). PCoA combined with PERMANOVA demonstrates that there are notable dissimilarities in the composition of fungal communities between CK, PF, and three mixed forests (MF1, MF2, and MF3) treatments (*p* = 0.001, Figure 2c). The Anosim analysis based on Bray–Curtis also reveals significant differences in soil fungal communities among the various treatments (R = 0.96, *p* = 0.001, Figure 2d). The soil fungal communities are primarily composed of Basidiomycota (34.29%), unclassified (31.84%), Ascomycota (25.52%), and Mucoromycota (8.27%) at the phylum level (Figure 2e). The relative abundance of Basidiomycota in MF1 and unclassified in MF3 is significantly higher than in the PF treatment group (*p* < 0.05, Figure 2e). At the genus level, taxa with relative abundances greater than 1% were higher in the MF1 treatment, with Tomentella and Lactifluus showing the highest relative abundances (Figure 2f).

### 3.3. Fungal Co-Occurrence Networks

**Figure 3:**
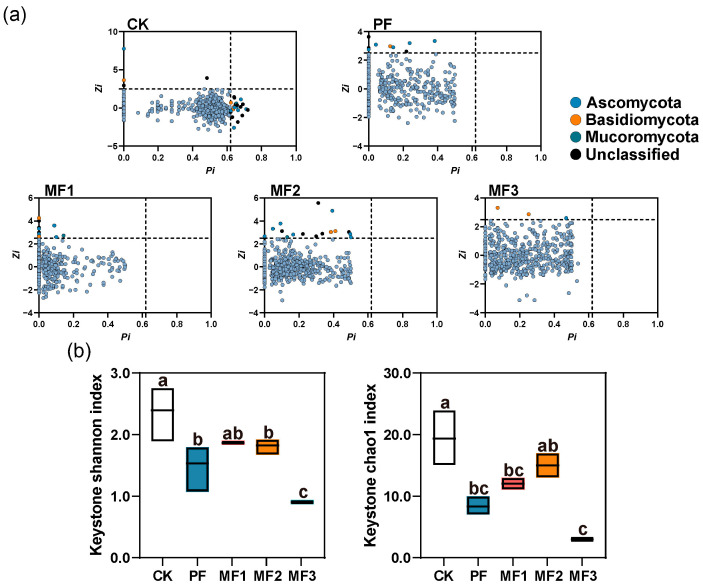
Zi–Pi plots of fungi (**a**) in different forest stands showing the distribution of key taxa based on topological interactions. The threshold values of Zi and Pi for categorizing ASVs were 2.5 and 0.62 respectively. (**b**) The diversity of fungal keystone taxa is indicated by the Shannon index and the Chao1 index. Nodes in the network can be classified into network hubs (Zi > 0.25, Pi > 0.62;), module hubs (Zi > 0.25, Pi ≤ 0.62), connectors (Zi ≤ 0.25, Pi > 0.62), and peripherals (Zi ≤ 0.25, Pi ≤ 0.62). Zi, the within-module connectivity; Pi, the among-module connectivity. Lowercase letters indicate the significant difference among treatments at *p* < 0.05. CK: evergreen broadleaf forest. PF: pure *Eucalyptus* forest. MF1: mixed *Eucalyptus* and *Castanopsis hystrix* forest. MF2: mixed *Eucalyptus* and *Michelia macclurei* forest. MF3: mixed *Eucalyptus* and *Magnolia hypolampra* forest.

We conducted a comparative analysis of the soil fungal co-occurrence networks among evergreen broadleaf forests, pure *Eucalyptus* plantations, and mixed *Eucalyptus*–broadleaf plantations (Figure S1). In the fungal networks, the proportion of positive correlations ranged from 45.55% to 60.46%, while negative correlations accounted for 39.54% to 54.45%. Network topological metrics revealed that Ascomycota, Basidiomycota, Mucoromycota, and a number of unclassified fungal ASVs were identified as network hubs (Figure 3a). A comparison of the diversity indices of key fungal taxa among the different treatments revealed that the evergreen broadleaf forests exhibited higher Shannon and Chao1 indices for key fungal taxa than both the pure *Eucalyptus* plantations and the mixed *Eucalyptus*–broadleaf plantations. Nevertheless, there was no statistically significant difference in the diversity of key fungal taxa between pure *Eucalyptus* plantations and mixed *Eucalyptus*–broadleaf plantations (*p* > 0.05, Figure 3b).

**Figure 4:**
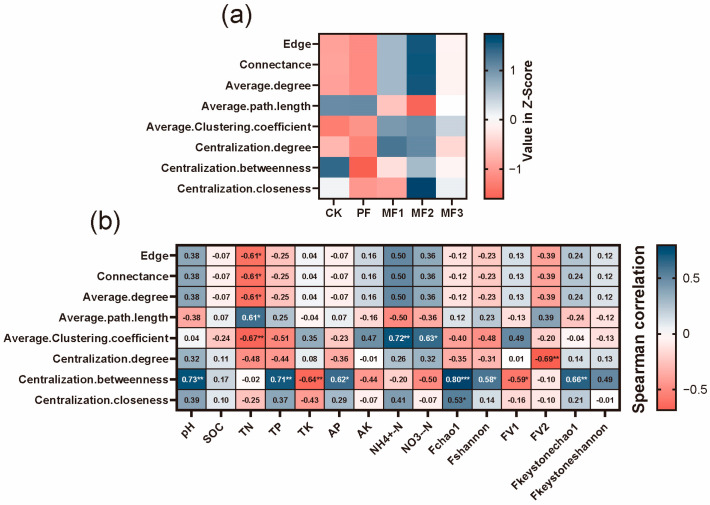
(**a**) Heatmaps of z-score values of topological properties of fungal co-occurrence networks in different treatments. (**b**) Correlation heatmap of topological properties of co-occurrence networks with soil properties and fungal diversity. * represents the *p*-value of the permutation test, and different symbols indicate different levels of significance: * *p* < 0.05, ** *p* < 0.01, *** *p* < 0.001. CK: evergreen broadleaf forest. PF: pure *Eucalyptus* forest. MF1: mixed *Eucalyptus* and *Castanopsis hystrix* forest. MF2: mixed *Eucalyptus* and *Michelia macclurei* forest. MF3: mixed *Eucalyptus* and *Magnolia hypolampra* forest.

Moreover, we conducted a comparative analysis of the topological properties of the co-occurrence networks, including edge, connectance, average degree, average path length, average clustering coefficient, centralization degree, centralization betweenness, and centralization closeness, across different treatments (Figure 4a). The results indicated that the soil fungal networks in mixed *Eucalyptus*–broadleaf plantations exhibited higher edge, connectance, average degree, average clustering coefficient, and node centrality compared to evergreen broadleaf forests and pure *Eucalyptus* plantations. Spearman’s correlation analysis demonstrated that the topological properties of the fungal networks exhibited a positive correlation with soil pH, NH_4_^+^-N, and NO_3_^−^-N (r = 0.38 to 0.72, Figure 4b) and a negative correlation with soil TN and total TP (r = 0.25 to 0.67, Figure 4b).

### 3.4. Relationship between Fungal Community Structure and Soil Properties

The results of the correlation heatmap between soil properties and fungal community diversity indicate that there is a significant positive relationship between soil pH and AP and the diversity of key fungal taxa (r = 0.54 to 0.78, *p* < 0.05, Figure S2). Additionally, a positive correlation was observed between fungal community diversity and soil TP and AP (r = 0.55 to 0.82, *p* < 0.05), while a negative correlation was evident between fungal community diversity and soil TK and nitrate nitrogen (r = 0.62 to 0.70, *p* < 0.05). Furthermore, the fungal β-diversity indices were found to be significantly positively correlated with soil TK, AK, NH_4_^+^-N, and NO_3_^−^-N (r = 0.75 to 0.88, *p* < 0.01) while exhibiting significant negative correlations with SOC and TN (r = 0.63 to 0.66, *p* < 0.05).

**Figure 5:**
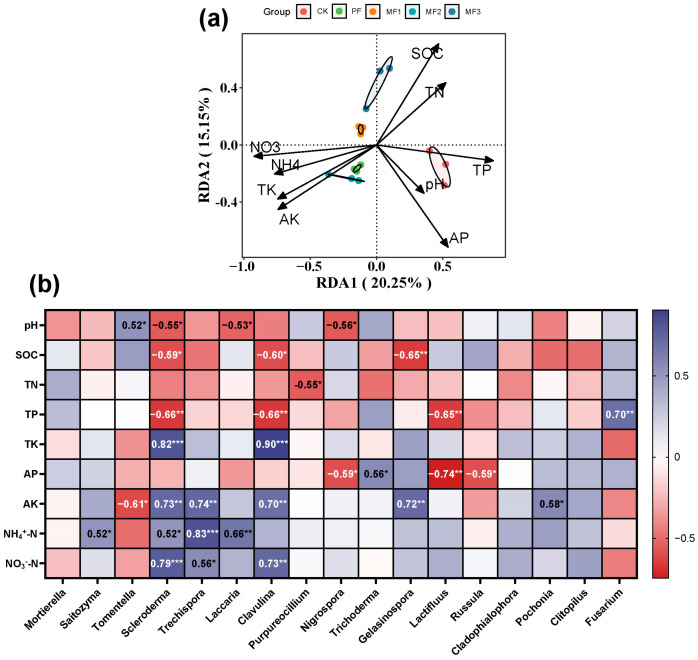
The relationship between soil properties and fungal communities was analyzed using (**a**) redundancy analysis and (**b**) Spearman correlation between soil properties and relative abundance at the level of fungal genus. * represents the *p*-value of the permutation test, and different symbols indicate different levels of significance: * *p* < 0.05, ** *p* < 0.01, *** *p* < 0.001. CK: evergreen broadleaf forest. PF: pure *Eucalyptus* forest. MF1: mixed *Eucalyptus* and *Castanopsis hystrix* forest. MF2: mixed *Eucalyptus* and *Michelia macclurei* forest. MF3: mixed *Eucalyptus* and *Magnolia hypolampra* forest.

The redundancy analysis (RDA) results indicated that soil SOC, TP, AP, AK, and NO_3_^−^-N were the most important environmental parameters influencing fungal communities, with the first and second axes explaining 20.25% and 15.15% of the variance, respectively (Figure 5a). The correlation heatmap between soil properties and the relative abundance of fungal genus revealed that the dominant genus Scleroderma and Clavulina exhibited significant positive associations with soil TK, AK, and NO_3_^−^-N but significant negative associations with SOC and TP. In addition, soil pH showed a significant negative correlation with Scleroderma, Laccaria, and Nigrospora, and AP showed a significant negative correlation with Nigrospora, Lactifluus, and Russula (Figure 5b).

### 3.5. Soil Multifunctionality and Driving Force

**Figure 6:**
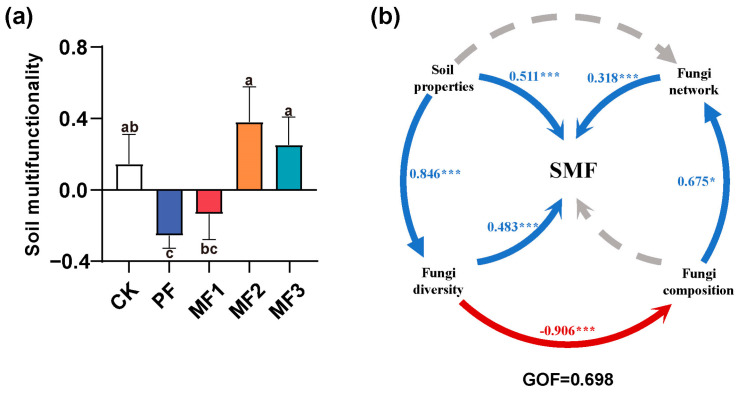
(**a**) Differences among soil multifunctionality (SMF) of different treatments. (**b**) Partial least squares path modeling (PLS-PM) indicating potential direct and indirect effects of soil properties and the fungal diversity, composition, and co-occurrence networks on SMF. Lowercase letters indicate the significant difference among treatments at *p* < 0.05. Blue arrows indicate positive correlations, red arrows indicate negative correlations, gray arrows indicate insignificant correlations, and numbers on the arrows indicate path coefficients. * *p* < 0.05; *** *p* < 0.001. R^2^ indicates the proportion of variance explained. Goodness-of-fit (GOF) provides a quantitative assessment of the overall fit quality of the model. CK: evergreen broadleaf forest. PF: pure *Eucalyptus* forest. MF1: mixed *Eucalyptus* and *Castanopsis hystrix* forest. MF2: mixed *Eucalyptus* and *Michelia macclurei* forest. MF3: mixed *Eucalyptus* and *Magnolia hypolampra* forest.

The one-way ANOVA revealed that the SMF in the MF2 and MF3 treatments was significantly higher than in the PF treatment (*p* < 0.05, Figure 6a). The random forest model demonstrated that soil AP, LAP, AG, ACP, the fungal Chao1 index, and the topological properties of fungal co-occurrence networks, specifically centralization betweenness and centralization closeness, were strong predictors of changes in SMF (9.25% to 15.35%, *p* < 0.05, Figure S3b). Spearman’s correlation analysis revealed a positive correlation between SMF and soil pH, TP, and AP (r = 0.44 to 0.49, Figure S3a) and negatively correlated with soil TK (r = 0.40). Furthermore, a significant positive correlation was observed between SMF and the concentrations of ACP, LAP, AG, and NAG (r = 0.60 to 0.79, *p* < 0.05, Figure S3a). Moreover, the fungal Chao1 index and the topological properties of fungal co-occurrence networks, including centralization betweenness and centralization closeness, demonstrated a significant positive correlation with SMF (r = 0.65 to 0.84, *p* < 0.05, Figure S3a).

The PLS-PM model was developed to further examine the direct and indirect impacts of soil properties, fungal community diversity, composition, and co-occurrence networks on SMF. The PLS-PM results indicated a significant positive correlation between SMF and the soil properties, fungal diversity, and topological properties of fungal co-occurrence networks (r = 0.318 to 0.511, *p* < 0.001, Figure 6b). Furthermore, soil properties exert an indirect influence on SMF through their impact on fungal diversity and composition, which in turn affect fungal community networks (r = 0.675 to 0.906, *p* < 0.05, Figure 6b). The results of the PLS-PM analysis align with those of the random forest model, indicating that the establishment of mixed *Eucalyptus*–broadleaf plantations could have a significant impact on SMF by modifying soil attributes and the composition of fungal communities (Figure 6b).

## 4. Discussion

### 4.1. Effects of Eucalyptus and Broadleaf Mixing on Soil Properties and Enzyme Activities

This study revealed notable discrepancies in soil characteristics and enzyme activities between mixed *Eucalyptus* and native broadleaf plantations and pure *Eucalyptus* plantations (*p* < 0.05, Table 1 and Figure 1). The difference tendencies among different mixed forests are not the same. In particular, the soil pH, SOC, NH_4_^+^-N, and NO_3_^−^-N contents were found to be significantly elevated in the mixed plantations. However, the observed differences in soil properties among tree species and *Eucalyptus* mixes may be attributed to the inherent biological characteristics of each tree species and their distinct interactions with *Eucalyptus* [10,45]. The mixing of different tree species with *Eucalyptus* results in varying effects due to their distinct living habits. Additionally, whether mixing with *Eucalyptus* can bring complementary effects and help construct a reasonable forest structure is also a factor [10]. Our findings corroborate those of previous studies, which have indicated that the majority of mixed *Eucalyptus* and broadleaf forests exert a beneficial influence on soil properties, enzyme activities, and productivity [46]. The presence of a diverse range of plant species in mixed plantations provides a variety of litter and root exudates, which serve as a rich source of nutrients for soil microbes [47]. The diversity of tree species has been demonstrated to enhance microbial activity and diversity, thereby promoting nutrient cycling and availability [48]. It has been demonstrated in studies that tree species diversity enhances soil microbial carbon use efficiency, which in turn increases the sequestration of carbon in forests [49]. The complex root systems observed in mixed plantations contribute to enhanced physical soil properties, including the formation of soil aggregates and increased porosity. Such enhancements serve to reinforce the forest’s resilience to drought conditions, thereby maintaining superior soil quality [50]. Moreover, our study demonstrated that mixed plantations effectively enhanced the activities of pivotal enzymes involved in organic carbon cycling, as well as nitrogen and phosphorus transformations, including Ure, LAP, AG, BG, CB, NAG, and PPO. Prior research has demonstrated that these extracellular enzymes are significantly correlated with soil pH, organic carbon concentration, and moisture content [51]. The implementation of mixed plantations can enhance soil pH, organic carbon content, aeration, and moisture retention, which may contribute to the elevated enzyme activities observed in our study.

### 4.2. Eucalyptus Broad Mixing Enhances Fungal Network Complexity

Microbial interactions and their functions can elucidate the ecological relationships that are influenced by resource availability and environmental heterogeneity [52]. The analysis of co-occurrence networks represents a valuable tool for elucidating the intricacies of the microbiome and the influence of microbial associations on ecosystem functions [17]. The fungal community in *Eucalyptus*–broadleaf mixed forests was found to exhibit higher edge, connectance, average degree, average path length, average clustering coefficient, centralization degree, centralization betweenness, and centralization closeness compared to pure *Eucalyptus* forests (Figure 4a). This suggests that mixing *Eucalyptus* with native broadleaf species effectively enhances the complexity and stability of fungal networks, thereby improving ecosystem stability and functionality. Moreover, mixed forests introduce diverse litter and root exudates [53], which provide a rich source of nutrients and create varied microenvironments, thus promoting microbial diversity and network complexity. The network complexity and multifunctionality of soil microbial communities are frequently vulnerable to environmental disturbances [52]. However, *Eucalyptus*–broadleaf mixed forests can effectively enhance soil nutrient levels and enzyme activities, thereby enabling microbes to decompose soil organic matter more efficiently and accelerate nutrient cycling. This, in turn, results in an increase in microbial diversity and interactions, leading to a higher level of microbial network complexity. Furthermore, research has demonstrated that soil pH is a principal determinant of microbial network complexity [54]. This corroborates our findings that mixing *Eucalyptus* with broadleaf species can effectively mitigate soil acidification issues associated with *Eucalyptus* monocultures and enhance fungal network complexity.

### 4.3. Soil Nutrients, Fungal Diversity, and Network Complexity Jointly Drive Soil Multifunctionality

This study found that SMF in mixed *Eucalyptus* and broadleaf plantations was significantly higher than in pure *Eucalyptus* plantations (*p* < 0.05, Figure 6a). We revealed significant positive correlations between soil properties (pH, TP, TK, and AP), fungal diversity, and the complexity of co-occurrence networks with SMF through PLS-PM analysis (*p* < 0.05, Figure 6b). Recently, there has been a notable increase in research on mixed *Eucalyptus* and broadleaf plantations [55]. Mixed forests play an essential role in enhancing soil nutrients and enzyme activities, soil aggregate proportion and stability, as well as other ecosystem functions [48]. This suggests that mixed planting patterns exhibit greater levels of ecological function than monocultures [56]. Mixed forests can develop layered stand structures, which facilitate the efficient utilization of forest space and environmental resources. They enhance light use, regulate the microclimate, boost forest productivity, increase species diversity, and promote ecological balance. [57]. Soil microbial communities play a pivotal role in the transformation and cycling of soil nutrients [16]. Compared to pure *Eucalyptus* plantations, mixed *Eucalyptus* and broadleaf plantations have higher soil fertility, leading to greater microbial activity and diversity. Additionally, the improved interaction networks among various soil microbial functional groups enhance nutrient availability and uptake by plants [58]. It has been demonstrated that more complex microbial networks and higher network connectivity are crucial for microbial community stability and ecosystem multifunctionality [59]. The maintenance of ecosystem functions is not the responsibility of individual species, but rather the result of interactions among different species [60]. The diverse and complex soil fungal communities present in mixed forests can mitigate the risks associated with environmental changes due to their greater number of species and interactions [15]. The intricate microbial networks enhance functional redundancy, allowing species with similar functions to compensate for losses and maintain ecosystem functionality under environmental stress [61]. For example, interactions among heterotrophic bacterial communities can increase the rates of organic carbon decomposition and nitrogen mineralization [62], and the associations between bacteria and fungi are closely linked to soil organic carbon cycling [8]. It can thus be concluded that the increased complexity of microbial networks implies enhanced functional connections, which in turn improve the efficiency of energy and material flow within the ecosystem. This has a positive impact on SMF. In conclusion, this study highlights the considerable benefits of mixed *Eucalyptus* and broadleaf plantations in enhancing SMF. By improving soil properties, increasing fungal diversity, and enhancing the complexity of co-occurrence networks, mixed forests can significantly enhance soil ecological functions and stability. These findings provide important references for future afforestation projects, recommending the incorporation of diverse tree species to enhance ecosystem carbon benefits and resilience.

## 5. Conclusions

Our research enhances the existing understanding of microbial diversity and ecosystem functioning, with a particular focus on the impact of mixed plantations on SMF. Our empirical evidence demonstrates that *Eucalyptus* combined with native broadleaf species can effectively improve soil properties, enzyme activities, and soil multifunctionality. Mixing *Eucalyptus* and broadleaf species can increase the fungal community diversity and complexity of the co-occurrence network. The results of the partial least squares model show that the soil properties, fungal community diversity, and complexity of the fungal co-occurrence network are all key factors driving changes in soil multifunctionality. The findings of this research provide compelling evidence for the connection between fungal communities and SMF in mixed *Eucalyptus*–broadleaf plantations. These findings underscore the significance of mixed plantations in promoting ecosystem health and functionality, particularly in terms of enhancing soil quality and biodiversity. Future studies should delve deeper into the long-term effects of various tree species combinations on soil ecosystems to optimize forest management and ecological restoration strategies.

## Figures and Tables

**Table 1:** Soil chemical properties of *Eucalyptus* broad mixed forest.

	CK	PF	MF1	MF2	MF3
pH	4.4 ± 0.09 a	4.08 ± 0.07 b	4.23 ± 0.06 ab	4.39 ± 0.27 a	4.15 ± 0.05 ab
SOC/(g/kg)	33.18 ± 3.3 ab	29.19 ± 1.37 bc	31.28 ± 0.77 bc	28.7 ± 1.11 c	36.14 ± 2.35 a
TN/(g/kg)	2.25 ± 0.11 a	2.15 ± 0.34 a	2.01 ± 0.1 ab	1.76 ± 0.01 b	2.33 ± 0.08 a
TP/(g/kg)	1.11 ± 0.12 a	0.56 ± 0.08 bc	0.48 ± 0.03 c	0.64 ± 0.16 bc	0.72 ± 0.08 b
TK/(g/kg)	4.6 ± 0.09 b	18.55 ± 1.49 a	16.27 ± 3.65 a	13.98 ± 3.82 a	5.16 ± 1.1 b
AP/(mg/kg)	35.48 ± 8.2 a	21.08 ± 1.34 b	9.85 ± 4.13 c	26.96 ± 0.55 ab	10.78 ± 4.21 c
AK/(mg/kg)	65.83 ± 6.37 c	100.54 ± 8.41 ab	82.27 ± 9.85 bc	107.13 ± 16.31 a	72.71 ± 6.43 c
NH_4_^+^-N/(mg/kg))	2.39 ± 0.23 c	4.5 ± 1.34 b	3.92 ± 0.2 b	6.85 ± 0.9 a	4.28 ± 0.4 b
NO_3_^−^-N/(mg/kg)	6.11 ± 0.84 c	22.1 ± 3.8 ab	22.4 ± 4.8 ab	22.94 ± 2.78 a	16.03 ± 2.95 b

Note: Values are mean ± standard error. Based on ANOVA, different lowercase letters indicate significant differences among different treatments (*p* < 0.05). pH: acidity and alkalinity; SOC: soil organic carbon; TN: total nitrogen; TP: total phosphorus; TK: total potassium; AP: available phosphorus; AK: available potassium; NH_4_^+^-N, ammonium nitrogen; NO_3_^−^-N, nitrate nitrogen; CK: evergreen broadleaf forest. PF: pure *Eucalyptus* forest. MF1: mixed *Eucalyptus* and *Castanopsis hystrix* forest. MF2: mixed *Eucalyptus* and *Michelia macclurei* forest. MF3: mixed *Eucalyptus* and *Magnolia hypolampra* forest.

## Data Availability

The raw data supporting the conclusions of this article will be made available by the authors on request.

## Supplementary Materials

The following supporting information can be downloaded at: https://www.mdpi.com/article/10.3390/jof10100709/s1, Figure S1: Co-occurrence network of fungi in the soil of different treatments; Figure S2: Heatmap of correlation between soil properties and fungal community diversity; Figure S3: (a) Heatmap of correlation between soil multifunctionality (SMF) and soil properties, enzyme activities, fungal community diversity, and topological properties of co-occurrence networks. (b) Random forest analysis showed the associations between SMF and soil properties, enzyme activities, fungal community diversity, and topological properties of co-occurrence networks.
