# Supplementary material for: Eucalyptus and Native Broadleaf Mixed Cultures Boost Soil Multifunctionality by Regulating Soil Fertility and Fungal Community Dynamics
Source: J Fungi (Basel). 2024 Oct 11;10(10):709. doi: 10.3390/jof10100709 (PMC11508252; doi:10.3390/jof10100709)
Supplement: Supplementary file 1 [file jof-10-00709-s001.zip › jof-3228299-supplementary.pdf]

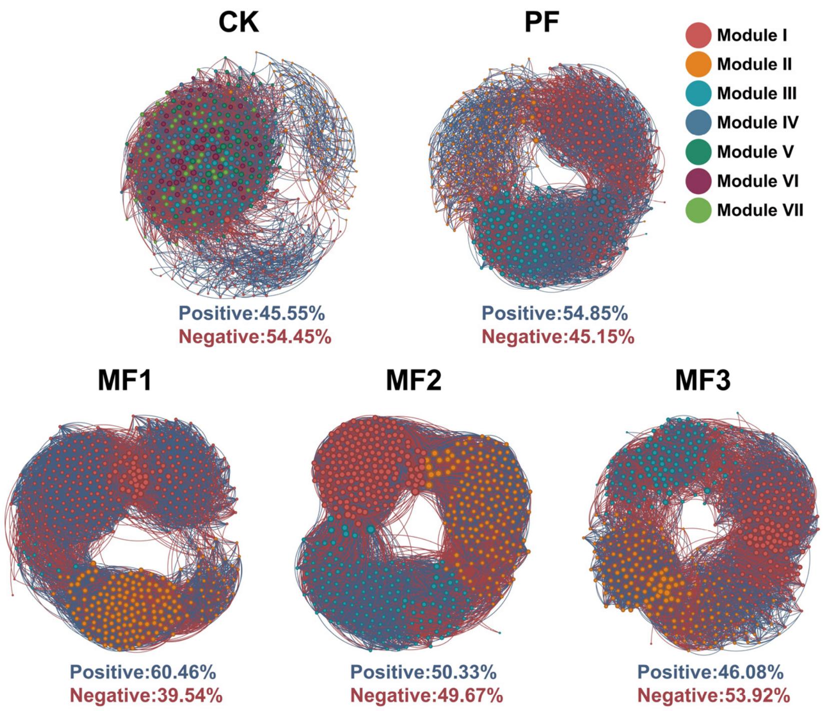

Figure S1 Co-occurrence network of fungi in the soil of different treatment. Each point in the graph represents a node in the network, the size of the point indicates the abundance of the node, the color indicates the module classification, and the color of the edge indicates the type of influence between the nodes: blue for positive influence, red for negative influence.

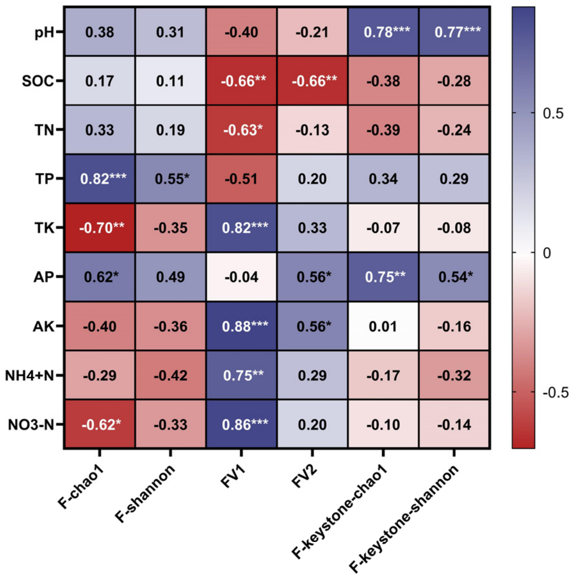

Figure S2 Heat map of correlation between soil properties and fungal community diversity. *

represents the P value of permutation test, different symbols indicate different levels of significance: * $p < 0.05$, ** $p < 0.01$, *** $p < 0.001$.

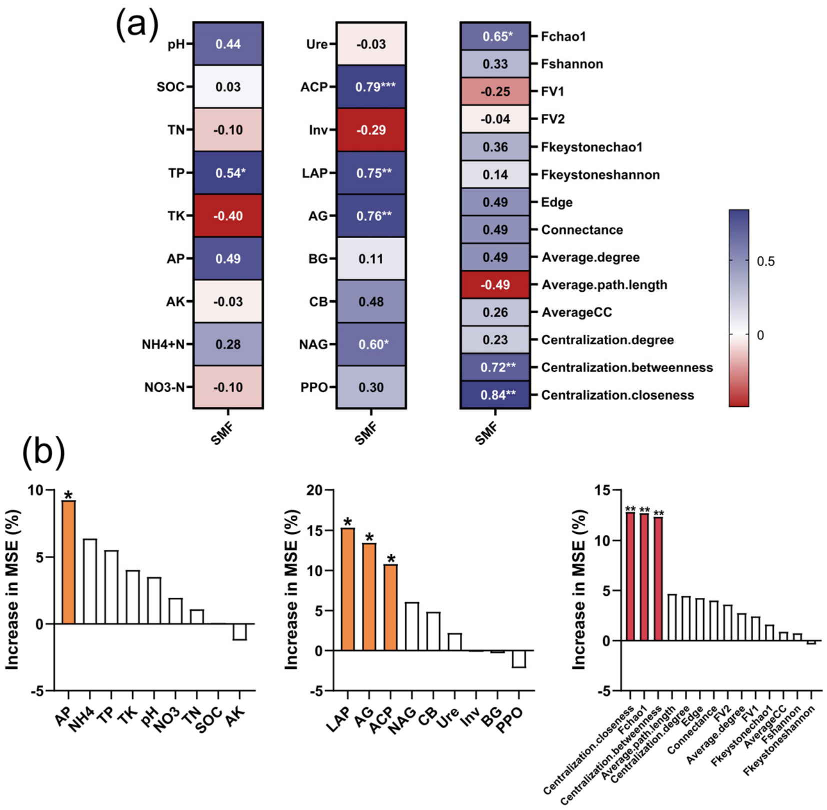

Figure S3 (a) Heatmap of correlation between soil multifunctionality (SMF) and soil properties, enzyme activities, fungal community diversity, and topological properties of co-occurrence networks. (b) Random forest analysis showed the associations between SMF and soil properties, enzyme activities, fungal community diversity, and topological properties of co-occurrence networks. * represents the P value of permutation test, different symbols indicate different levels of significance: * $p < 0.05$, ** $p < 0.01$, *** $p < 0.001$.
